# Developments in continuous therapy and maintenance treatment approaches for patients with newly diagnosed multiple myeloma

**DOI:** 10.1038/s41408-020-0273-x

**Published:** 2020-02-13

**Authors:** Meletios A. Dimopoulos, Andrzej J. Jakubowiak, Philip L. McCarthy, Robert Z. Orlowski, Michel Attal, Joan Bladé, Hartmut Goldschmidt, Katja C. Weisel, Karthik Ramasamy, Sonja Zweegman, Andrew Spencer, Jeffrey S. Y. Huang, Jin Lu, Kazutaka Sunami, Shinsuke Iida, Wee-Joo Chng, Sarah A. Holstein, Alberto Rocci, Tomas Skacel, Richard Labotka, Antonio Palumbo, Kenneth C. Anderson

**Affiliations:** 1 Hematology & Medical Oncology, Department of Clinical Therapeutics, National and Kapodistrian University of Athens, School of Medicine, Athens, Greece; 2 University of Chicago Medical Center, Chicago, IL USA; 3 Roswell Park Comprehensive Cancer Center, Buffalo, NY USA; 4 Department of Lymphoma/Myeloma, The University of Texas MD Anderson Cancer Center, Houston, TX USA; 5 Hematology Department, University Hospital Purpan, Toulouse, France; 6 Hematology Department, Hospital Clinic, Institut de Investigacions Biomediques August Pi I Sunyer (IDIBAPS), Barcelona, Spain; 7 Department of Internal Medicine V, University Medical Hospital and National Center of Tumor Diseases, University of Heidelberg, Heidelberg, Germany; 8 Department of Oncology, Hematology and Bone Marrow Transplantation with Section of Pneumology, University Medical Center Hamburg-Eppendorf, Hamburg, Germany; 9 Oxford University Hospitals, NHS Foundation Trust, Oxford, UK; 10 Department of Hematology, Cancer Center Amsterdam, Amsterdam University Medical Center, VU University Amsterdam, Amsterdam, The Netherlands; 11 Malignant Haematology and Stem Cell Transplantation Service, Alfred Health-Monash University, Melbourne, Australia; 12 National Taiwan University, Taipei, Taiwan; 13 Department of Hematology, Peking University People’s Hospital and Peking University Institute of Hematology, Beijing, China; 14 Department of Hematology, National Hospital Organization Okayama Medical Center, Okayama, Japan; 15 Department of Hematology and Oncology, Nagoya City University Graduate School of Medical Sciences, Nagoya, Japan; 16 Department of Haematology-Oncology, National University Cancer Institute, National University Health System, and Cancer Science Institute of Singapore, National University of Singapore, Singapore, Singapore; 17 Department of Internal Medicine, University of Nebraska Medical Center, Omaha, NE USA; 18 Department of Haematology, Manchester University Hospitals NHS Foundation Trust, Manchester, UK; 19 Faculty of Biology, Medicine and Health, School of Medical Science, Division of Cancer Science, University of Manchester, Manchester, UK; 20 Millennium Pharmaceuticals, Inc., a wholly owned subsidiary of Takeda Pharmaceutical Company Limited, Cambridge, MA USA; 21 Dana-Farber Cancer Institute, Boston, MA USA

**Keywords:** Cancer therapy, Myeloma, Cancer therapy, Myeloma, Therapeutics

## Abstract

The evolving paradigm of continuous therapy and maintenance treatment approaches in multiple myeloma (MM) offers prolonged disease control and improved outcomes compared to traditional fixed-duration approaches. Potential benefits of long-term strategies include sustained control of disease symptoms, as well as continued cytoreduction and clonal control, leading to unmeasurable residual disease and the possibility of transforming MM into a chronic or functionally curable condition. “Continuous therapy” commonly refers to administering a doublet or triplet regimen until disease progression, whereas maintenance approaches typically involve single-agent or doublet treatment following more intensive prior therapy with autologous stem cell transplant (ASCT) or doublet, triplet, or even quadruplet induction therapy. However, the requirements for agents and regimens within these contexts are similar: treatments must be tolerable for a prolonged period of time, should not be associated with cumulative or chronic toxicity, should not adversely affect patients’ quality of life, should ideally be convenient with a minimal treatment burden for patients, and should not impact the feasibility or efficacy of subsequent treatment at relapse. Multiple agents have been and are being investigated as long-term options in the treatment of newly diagnosed MM (NDMM), including the immunomodulatory drugs lenalidomide and thalidomide, the proteasome inhibitors bortezomib, carfilzomib, and ixazomib, and the monoclonal antibodies daratumumab, elotuzumab, and isatuximab. Here we review the latest results with long-term therapy approaches in three different settings in NDMM: (1) maintenance treatment post ASCT; (2) continuous frontline therapy in nontransplant patients; (3) maintenance treatment post-frontline therapy in the nontransplant setting. We also discuss evidence from key phase 3 trials. Our review demonstrates how the paradigm of long-term treatment is increasingly well-established across NDMM treatment settings, potentially resulting in further improvements in patient outcomes, and highlights key clinical issues that will need to be addressed in order to provide optimal benefit.

## Introduction

Outcomes in patients with multiple myeloma (MM) have improved substantially over the past two decades^1^. Ongoing increases in progression-free (PFS) and overall survival (OS) are being seen with novel regimens across treatment settings, associated with the evolving paradigm of long-term treatment approaches, including continuous therapy and maintenance, which can prolong disease control and improve PFS and sometimes OS compared to fixed-duration approaches^2,3^. Definitions of therapeutic approaches within this paradigm of long-term treatment are summarized in Table 1 ^4,5^.

**Table 1:** Definitions of therapeutic approaches within the paradigm of long-term treatment.

Continuous therapy	Maintenance therapy
•Commonly refers to administering a regimen until disease progression	•Commonly refers to treatment that differs from previous, more intensive therapy
•Typically a doublet or triplet, such as standard-of-care Rd^4^	•Typically single-agent or doublet therapy following ASCT, per the recent approval of single-agent lenalidomide^5^, or following doublet, triplet, or even quadruplet remission induction therapy

*ASCT* autologous stem cell transplant, *Rd* lenalidomide-dexamethasone.

This paradigm is being increasingly followed, with safety profiles of newer drugs improving long-term treatment feasibility vs. older agents^2^. Various long-term approaches in newly diagnosed MM (NDMM) are discussed within current guidelines and recommendations^1,6–10^. Consequently, and associated with benefits demonstrated in randomized clinical trials, long-term therapy is used extensively in routine clinical practice in some geographies. Maintenance was used in 81% of autologous stem cell transplant (ASCT) patients and 68% of nontransplant patients in 2017 US physician-reported data^11^. However, retrospective data on real-world practice patterns in Europe indicated only 12% of patients received maintenance as part of frontline treatment (acknowledging that this 2016 publication preceded the 2017 approval of lenalidomide in this setting)^12^.

We review the increasing importance of continuous therapy and maintenance in targeting the goal of improving outcomes and providing “functional cure” (i.e. long-term molecular remission^1,13^) in MM. We focus on long-term therapy in three settings: (1) maintenance treatment post ASCT; (2) continuous frontline therapy in nontransplant patients; (3) maintenance treatment post-frontline therapy in nontransplant patients. We highlight the latest evidence from phase 3 trials, plus emerging real-world data. We also consider practical requirements of long-term therapeutic approaches, including patient preferences and quality of life (QoL), tolerability and safety challenges, and pharmacoeconomics.

## Requirements/goals of long-term treatment

Requirements for long-term treatment approaches are summarized in Table 2 ^9,14–17^. The key goals of long-term treatment are to prolong disease control and improve PFS and OS. Among the potential benefits are suppression of clonal evolution (recognizing that emergence of drug-resistant clones is also a potential risk that could limit future treatment options)^18^; however, this hypothesis needs demonstrating in randomized controlled trials and is currently based on expert assumptions. Similarly, other potential benefits include sustained control of disease symptoms, immune modulation, and continued cytoreduction leading to unmeasurable residual disease—optimally, complete eradication of MM cells^2^. Deepening of response is an important goal, as deeper responses^19^ (and sustained deep response^20^) are associated with improved outcomes. Converting patients to, and sustaining, minimal residual disease (MRD)-negative status represents a step towards “functional cure”^13^. Emerging data from continuous therapy and maintenance approaches have already demonstrated a positive impact on rates of MRD-negative disease status^21–25^.

**Table 2:** Key requirements for long-term treatment approaches.

Requirement	Specific needs for continuous therapy and maintenance treatment
Efficacy/effectiveness	•Agents/regimens must be active. •Further long-term treatment options are needed that are efficacious across patient subgroups, including those with high-risk disease^17^, for whom longer-term treatment is a particular requirement to achieve sustained disease control. •Additional options are also needed that have demonstrated real-world feasibility and effectiveness, with no impact on feasibility or efficacy of subsequent treatment at relapse. •Given the heterogeneity of MM, long-term treatments incorporating multiple drugs with differing mechanisms of action may be required for prolonged disease control in specific patient subgroups^9^.
Tolerability/safety	•Must be able to be tolerated for a prolonged period with little-to-no cumulative or chronic toxicity or substantive adverse impact on patients’ QoL.
Minimal treatment burden	•Minimal treatment burden through convenience of administration is important, highlighting the preference for all-oral treatment options that avoid the patient and caregiver burden associated with repeat parenteral administration. •Indeed, patient preference for all-oral vs. injectable proteasome inhibitor-based treatment has been reported in the relapsed/refractory setting^14^. •All-oral regimens have been shown to have lower economic burden of illness, less activity impairment, lower productivity loss, and a trend towards greater convenience than injectable regimens in the frontline setting^15,16^. •A minimal treatment and toxicity burden is also important in the context of patients potentially otherwise preferring a treatment-free interval.

*QoL* quality of life.

## Post-ASCT maintenance therapy

Key phase 3 data on agents investigated as post-ASCT maintenance therapy are summarized in Table 3.

**Table 3:** Summary of data from key phase 3 studies/meta-analyses reporting comparative data on post-ASCT maintenance.

Study	Treatment (maintenance dose/duration)	*N*	Follow-up	DoT	Key efficacy outcomes	Key safety and tolerability data
Myeloma IX^26^	T (50–100 mg/day to PD) vs. no maintenance post ASCT	245 vs. 247	38 months^a^	9 months	Median PFS: 30 vs. 23 months (HR 1.42) 3-year OS: 75% vs. 80%	Discontinuation due to AEs: 52.2%^a^ Serious adverse reaction: 8.5%
	Meta-analysis, T (various doses/durations) vs. no T maintenance, inc. non-ASCT	1098 vs. 1333	NR	NR	OS: HR 0.75 7-year OS: 12.3% difference in rate, in favor of T maintenance	NR
HOVON-50^27,29^	TAD-ASCT-T (50 mg/day to PD) vs. VAD-ASCT-IFN	268 vs. 268	Initial analysis: 52 months Follow-up: 129 months	NR	Median EFS: 34 vs. 22 months (HR 0.60); HR 0.62 at follow-up Median PFS: 34 vs. 25 months (HR 0.67) OS: HR 0.96 OS from relapse: 20 vs. 31 months (HR 1.50)	T maintenance: Discontinuation due to AEs: 33%; 42% at follow-up (vs. 27% IFN) Grade 1/2/3/4 PN: 21/33/9/1%
NCIC-CTG Myeloma 10^28^	TP (T 200 mg/day, P 50 mg Q2d; up to 4 yrs) vs observation post ASCT	166 vs. 166	4.1 years	16.1 vs. 14.9 months	4-year PFS: 32% vs. 14% (HR 0.55) 4-year OS: 68% vs. 60% (HR 0.77) Median OS post-relapse: 27.7 vs. 34.1 months	Grade 3/4 thromboembolism: 7.3% vs. 0 Grade 3/4 sensory PN: 9.6% vs. 1.2%
IMWG meta-analysis, six studies^30^	T (various doses/durations) vs. no T maintenance, inc. non-ASCT	1276 vs. 1510	NR	NR	PFS: HR 0.65 OS: HR 0.84	NR
CALGB 100104^33–35^	R (10 mg/day to PD) vs. placebo post ASCT	231 vs. 229	Initial report: 34 months	NR	Median PFS/TTP: 46 vs. 27 months (HR 0.48) 3-year OS: 88% vs. 80% (HR 0.62)	Grade 3/4 AEs: 32%/16% vs. 12%/5% Grade 3/4 neutropenia: 32%/13% vs. 12%/3% Discontinuation due to AEs: 10%
			Follow-up: 91 months	31.0 vs. 18.1 months	Median PFS/TTP: 57.3 vs. 28.9 months (HR 0.57) Median OS: 113.8 vs. 84.1 months (HR 0.61) Median OS post-relapse: 42.6 vs. 39.2 months (HR 0.83)	Grade 3/4 neutropenia: 50% vs. 18% Discontinuation due to AEs: 18% Heme/solid/non-invasive SPMs: 8%/6%/5% vs. 1%/4%/3%
	R (10 mg/day to PD) vs. placebo post ASCT, adjusted for crossover	76 placebo patients crossed over to R	Updated: >91 months	NR	Median OS: ITT, unadjusted: 111.01 vs. 80.26 months, HR 0.61 RPSFTM adjustment for crossover: 111.01 vs. 70.96 months, HR 0.52	NR
IFM2005-02^36^	R (10–15 mg/day to PD) vs. placebo post ASCT	307 vs. 307	45 months	NR	≥ VGPR (randomization-to-post-maintenance): 61−84% vs. 59−76% 4-year PFS: 43% vs. 22% (HR 0.50) 4-year OS: 73% vs. 75% (HR 1.06)	Grade 3/4 hematologic AEs: 58% vs. 23% (neutropenia 51% vs. 18%) Discontinuation due to AEs: 27% vs. 15% SPMs: 3.1 vs. 1.2 ppy^a^100
GIMEMA RV-MM-PI-209^37^	R (10 mg, d 1–21, 28-d cycles, to PD) vs. no maintenance post-MPR (*n* = 132) or ASCT (*n* = 141) consolidation	126 vs. 125 (67 vs. 68 post ASCT)	51.2 months from enrollment	NR	ASCT-R vs. ASCT: Median PFS: 54.7 vs. 37.4 months 5-year OS: 78.4% vs. 66.6% R maintenance CR rate improvement: 15.7 to 35.7% R vs. no maintenance, post-MPR/ASCT Median PFS: 41.9 vs. 21.6 months (HR 0.47) OS: HR 0.64	R vs. no maintenance, post-MPR/ASCT Grade 3/4 AEs: Neutropenia 23.3% vs. 0% Infections 6.0% vs. 1.7% Dermatologic events 4.3% vs. 0% Discontinuation due to AEs: 5.2% vs. 0%
Phase 3 meta-analysis (above three trials)^32^	R (doses as per above three trials) vs. placebo/no maintenance post ASCT	605 vs. 603	79.5 months	Mean 28 vs. 22 months	Median PFS: 52.8 vs. 23.5 months (HR 0.48) Median PFS2: 73.3 vs. 56.7 months (HR 0.72) 7-year OS: 62% vs. 50% (HR 0.75)	Discontinuation due to AEs: 29.1% vs. 12.2% Heme/solid SPMs prior to PD: 5.3%/5.8% vs. 0.8%/2.0%
Myeloma XI^25,40^	R (10/25 mg, d 1–21, 28-d cycles, to PD) vs. observation post ASCT	730 vs. 518	31 months^b^	18 cycles (4-week cycles)^b^	Cumulative rate of response improvement at 60 months: 15.8% vs. 11.0% Median PFS: 57 vs. 30 months (HR 0.48) Median PFS2: not reached vs. 59 months (HR 0.57) 3-year OS: 87.5% vs. 80.2% (HR 0.69)	Grade 3/4 neutropenia: 28%/5%^b^ Discontinuations due to AEs: 28%^b^ SPMs: 5.3% vs. 3.1%^b^
NCT01091831^41^	RP (R 10 mg, d 1–21, 28-d cycles; P 50 mg, Q2d; to PD) vs. R alone post ASCT	60 vs. 57	41.0 vs. 42.3 months^c^	Median 28.9 vs. 25.3 months^c^	Data from enrollment (including ASCT): Median PFS: 37.6 vs. 31.5 months 4-year OS: 77% vs. 75%	Grade 3/4 AEs:^c^ Neutropenia: 8% vs. 13% Infections: 8% vs. 5% Discontinuations due to AEs: 5% vs. 8%
GMMG-MM5^60^	R (10–15 mg/d) → 2 yrs vs. R (10–15 mg/d) → CR post-PAD/VCD + ASCT	PAD-R → 2 yrs vs. VCD-R → 2 yrs vs. PAD-R → CR vs. VCD–R → CR: 125 vs. 126 vs. 126 vs. 125	60.1 months	74% vs. 75% vs 39% vs. 50% received maintenance 35% vs. 35% vs 14% vs. 18% completed 2 years	Median PFS: 43.2 vs. 40.9 vs. 35.9 vs. 35.7 months 36-month OS: 83% vs. 85% vs 75% vs. 77%	Rates of grade ≥ 3 AEs and grade ≥ 2 infections, cardiac disorders, neuropathy, and thromboembolic events: 87.3% vs. 91.3% vs. 79.5% vs. 77.4% AEs during maintenance (R → 2 years vs. R → CR): 77.6% vs. 58.2% Grade ≥ 2 infections (R → 2 years vs. R → CR): 52.7% vs. 32.3%
HOVON-65/GMMG-HD4^52,53^	PAD-ASCT-V (1.3 mg/m^2^ IV, Q2w, up to 2 yrs) vs. VAD-ASCT-T (50 mg/day, up to 2 yrs)	413 vs. 414 (270 vs. 230 maintenance)	Initial analysis: 41 months	47% vs. 27% received 2 years of maintenance	Response improvement during maintenance: 23% vs. 24% Median PFS: 35 vs. 28 months (HR 0.75) Median PFS from last ASCT: 31 vs. 26 months 5-year OS: 61% vs. 55% (HR 0.81)	During maintenance: Grade 3/4 AE: 48% vs. 46% Grade 3/4 infections: 24% vs. 18% New-onset grade 3/4 PN: 5% vs. 8% Discontinuation due to toxicity: 11% vs. 30%
			Updated analysis: 96 months	50% vs. 28% received 2 years of maintenance	Median PFS: 34 vs. 28 months (HR 0.76) Median OS: 91 vs. 82 months (HR 0.89) Median OS from relapse: 43 vs. 40 months (HR 1.02)	SPMs: 7% vs. 7%
GEM05MENOS65^54^	VT (V 1.3 mg/m^2^ IV, d 1, 4, 8, 11, Q3M; T 100 mg/day) vs. T (T 100 mg/day) vs. IFN (3 MU × 3 per week) post ASCT, for up to 3 yrs	91 vs. 88 vs. 92	58.6 months	2.05 vs. 1.6 vs. 1.55 years	Improvement in CR rate: 21% vs. 11% vs. 17% Median PFS: 50.6 vs. 40.3 vs. 32.5 months 5-year OS: 78% vs. 72% vs. 70%	Grade 2−3 PN: 48.8% vs. 34.4% vs. 1% Discontinuation due to toxicity: 21.9% vs. 39.7% vs. 20%
TOURMALINE-MM3^58^	Ixazomib (3–4 mg, d 1, 8, 15, 28-d cycles; up to 2 yrs) vs. placebo post ASCT	395 vs. 261	31 months	25 vs. 22 4-week cycles	Response improvement: 46% vs. 32% (RR 1.41) Median PFS: 26.5 vs. 21.3 months (HR 0.72)	Grade ≥ 3 AEs: 42% vs. 26% Grade ≥ 3 infections/infestations: 15% vs. 8% Grade ≥ 3 GI disorders: 6% vs. 1% Discontinuation due to AEs: 7% vs. 5%

*AE* adverse event, *ASCT* autologous stem cell transplant, *CR* complete response, *d* day(s), *DoT* duration of treatment, *EFS* event-free survival, *GI* gastrointestinal, *heme* hematologic, *HR* hazard ratio, *IMWG* International Myeloma Working Group, *IFN* interferon, *inc.* including, *IV* intravenous, *MPR* melphalan-prednisone-lenalidomide, *MU* million units, *NR* not reported, *OS* overall survival, *P* prednisone, *PAD* bortezomib-doxorubicin-dexamethasone, *PD* progressive disease, *PFS* progression-free survival, *PFS2* progression-free survival from start of treatment to progression on next line of treatment, *PN* peripheral neuropathy, ppy*100 per 100 patient-years, *Q2d* every other day, *Q2w* every 2 weeks, *Q3M* every 3 months, *R* lenalidomide, *RP* lenalidomide-prednisone, *RPSFTM* rank-preserving structural failure time model, *RR* relative risk, *SPMs* second primary malignancies, *T* thalidomide, *TAD* thalidomide-doxorubicin-dexamethasone, *TP* thalidomide-prednisone, *TTP* time to progression, *V* bortezomib, *VAD* vincristine-doxorubicin-dexamethasone, *VGPR* very good partial response, *VT* bortezomib-thalidomide, *wk* week, *yrs* years.

^a^Data shown for all 408 vs. 410 patients randomized to maintenance, not just post-ASCT intensive pathway.

^b^Overall data in 1137 vs. 834 patients randomized to lenalidomide vs. observation across both the transplant-eligible and transplant-ineligible pathways.

^c^Overall data in 117 vs. 106 patients randomized to lenalidomide-prednisone vs. lenalidomide maintenance across the CRD and ASCT consolidation arms.

### Immunomodulatory drugs

Thalidomide maintenance has been studied in multiple phase 3 trials^26–29^ and meta-analyses^26,30^, which generally showed a significant PFS benefit; a meta-analysis by the International Myeloma Working Group (IMWG) demonstrated a 35% reduction in risk of progression or death^30^. However, less uniform findings have been reported regarding OS, with a significant benefit not found in the majority of individual studies but an overall significant improvement seen in the IMWG (hazard ratio (HR) 0.84)^30^ and Myeloma IX-related (HR 0.75)^26^ meta-analyses. Importantly, in some studies, limited durations of thalidomide maintenance and high discontinuation rates due to toxicity were reported^26,27,29^, as well as poorer survival following disease progression among patients exposed to thalidomide maintenance^28^, suggesting the selection of more resistant clones^18^. Thalidomide (vs. no maintenance) was associated with no PFS benefit and an adverse impact on OS in patients with high-risk cytogenetic abnormalities in the Myeloma IX trial (median OS 35 vs. 47 months)^26,31^. Thalidomide is not approved as post-ASCT maintenance.

Multiple phase 3 studies of single-agent lenalidomide as post-ASCT maintenance have been reported (Table 3), with the meta-analysis^32^ of 1208 patients who received lenalidomide vs. placebo/no maintenance post ASCT in the CALGB 100104 ^33–35^, IFM2005-02 ^36^, and GIMEMA RV-MM-PI-209 ^37^ studies resulting in its approval in this setting (Table 3)^5,32^. These studies showed substantial PFS benefit with lenalidomide vs. placebo/observation (HR 0.47–0.57), and significant OS improvements were reported in the CALGB and GIMEMA studies but not in IFM2005-02. In addition to early termination of maintenance due to a second primary malignancy (SPM) signal^36^, the fact that all patients in IFM2005-02 received lenalidomide consolidation post ASCT and that maintenance was not continued until progression may have contributed to the disparate OS findings. Importantly, in contrast to thalidomide, median OS post-relapse in CALGB 100104 appeared similar in the lenalidomide and placebo groups^33,34^. This is supported by recent reports showing lenalidomide maintenance resulting in prolonged time to disease progression on subsequent treatment (PFS2) and having no adverse impact on post-relapse survival^38^, including in patients receiving subsequent immunomodulatory-drug-based therapies^38,39^.

Subgroup analyses from the meta-analysis of lenalidomide maintenance demonstrated a uniform PFS benefit (HRs 0.40–0.58) vs. placebo/no maintenance in patients regardless of age, disease stage, and post-ASCT response, although limited benefit was reported in some high-risk subgroups (renal impairment post ASCT, HR 0.79; elevated lactate dehydrogenase, HR 0.89; adverse-risk cytogenetics, HR 0.86)^32^. However, incomplete data across studies precluded any definitive statement. OS findings were disparate, with no benefit seen in patients with stage III disease (HR 1.06), elevated lactate dehydrogenase (HR 1.17), or adverse-risk cytogenetics (HR 1.17). Additionally, OS benefit appeared more pronounced in patients achieving complete response (CR) or very good partial response (VGPR; HR 0.70) vs. <VGPR post ASCT (HR 0.88), and in patients who received lenalidomide-containing (HR 0.50) vs. non-lenalidomide (HR 0.82) induction^32^. The benefits of lenalidomide maintenance vs. observation in terms of PFS, PFS2, and OS have also been reported from the transplant-eligible intensive pathway of the Myeloma XI trial, with significant improvements observed (Table 3)^25,40^. This study had more patients with complete cytogenetic data and demonstrated improved PFS and OS with lenalidomide maintenance regardless of cytogenetic status, although absolute outcomes were poorer in high-risk patients^25^. Notably, median PFS improved by ~16 (ultra-high-risk patients) and ~31 months (high-risk patients) with lenalidomide vs. observation^25^.

The value of lenalidomide alone or in combination as maintenance has been demonstrated in other key studies (Table 3)^41^. In the IFM 2009 study, lenalidomide maintenance for 1 year following bortezomib-lenalidomide-dexamethasone (VRd) induction plus ASCT vs. prolonged VRd increased the ≥VGPR rate (78% vs. 69% to 85% vs. 76%, respectively)^42^. Similarly, an ongoing phase 2 study of lenalidomide-elotuzumab as post-ASCT maintenance showed response improvements in 33% of patients, with 20% converting to CR^43^. Additionally, while some patients (4–7%^44,45^) may convert to MRD-negative status post ASCT without requiring maintenance, analyses of studies employing lenalidomide maintenance, including Myeloma XI, EMN02/HO95, and RV-MM-EMN-441, have demonstrated substantially higher rates of conversion from MRD-positive to MRD-negative status of ~27–48%^24,44,46^.

Importantly, the value of lenalidomide maintenance is being demonstrated in the real-world setting. Reports from the Connect® MM registry, a US noninterventional, prospective registry incorporating >3000 NDMM patients from 250 academic-, government-, and community-based centers, have highlighted that lenalidomide maintenance post ASCT results in improved PFS (median 54.5 vs. 30.4 months, HR 0.58) and OS (3-year rate: 85% vs. 70%; HR 0.45) vs. no maintenance^47,48^, and that maintenance has no adverse impact on QoL^49^. Real-world analyses from the Mayo Clinic (lenalidomide vs. no maintenance: median PFS 37 vs. 28 months, HR 0.48)^50^ and the Princess Margaret Cancer Centre in Toronto (median PFS 41.7 months with lenalidomide maintenance)^51^ have reflected efficacy findings from clinical trials, but at the cost of some tolerability, with 17%^50^ and 13% of patients^51^, respectively, discontinuing due to toxicity, and 70% requiring dose reductions in the Toronto study^51^.

### Proteasome inhibitors

Bortezomib-based maintenance post ASCT has been evaluated in two key phase 3 studies. In the HOVON-65/GMMG-HD4 study^52,53^, single-agent bortezomib maintenance for 2 years following bortezomib-based induction and ASCT contributed to improved response rates and outcomes vs. single-agent thalidomide maintenance for 2 years following vincristine-doxorubicin-dexamethasone induction and ASCT (Table 3); however, the isolated benefit of bortezomib vs. thalidomide maintenance was not entirely clear as patients were not re-randomized post ASCT. Bortezomib maintenance was better tolerated, with 11% of patients discontinuing due to toxicity vs. 30% with thalidomide; however, 13% of patients discontinued prior to bortezomib maintenance due to toxicity, primarily polyneuropathy. In the GEM05MENOS65 study^54^, patients were randomized to one of three induction regimens and then re-randomized to compare post-ASCT maintenance for ≤3 years with bortezomib-thalidomide (VT), thalidomide alone, or interferon. VT maintenance resulted in the greatest improvement in CR rate and the longest PFS, but OS was similar in all three maintenance arms (Table 3).

In HOVON-65/GMMG-HD4, long-term bortezomib-based treatment appeared to abrogate the poor prognostic impact of del(17p), with 8-year OS rates of 52% vs. 54% in patients with and without this cytogenetic abnormality, which was not a stratification factor^52^. However, the poor prognostic impact of other high-risk cytogenetic abnormalities—t(4;14) and gain 1q21—was not overcome^52^. A recent analysis proposed that this was associated with additional subclonal heterogeneity^55^, suggesting the need for combination continuous therapy strategies in such high-risk patients. One single-center analysis has suggested that VRd consolidation and maintenance post ASCT may be promising for patients with high-risk disease (del17p, del1p, t(4;14), t(14;16); 96% ≥ VGPR, median PFS 32 months, 3-year OS 93%)^56^. Additionally, a phase 2 study has evaluated intensive bortezomib-based triplet therapy as post-ASCT maintenance in elderly patients, including 40% with high-risk disease, with promising early findings^57^. Notably, bortezomib maintenance post ASCT has demonstrated a PFS benefit (median 28 vs. 16 months) in high-risk patients in the real-world setting, in a retrospective, single-center analysis at Mayo Clinic^50^. However, the role of bortezomib-based therapy solely as maintenance cannot be extrapolated from these studies, in which patients may have also received bortezomib-based induction.

The recent phase 3 TOURMALINE-MM3 study showed, for the first time, the benefit of a proteasome inhibitor vs. placebo as post-ASCT maintenance, with the oral proteasome inhibitor ixazomib demonstrating a statistically significant PFS benefit (median 26.5 vs. 21.3 months, HR 0.72; HR 0.62 in 115 patients with high-risk cytogenetics) and a significantly greater rate of response improvement vs. placebo (Table 3)^58^. Ixazomib maintenance was planned for up to 2 years; 50% of patients completed the maximum duration, with 7% discontinuing due to toxicity and 36% due to progressive disease. With a median follow-up of 31 months, PFS2 and OS data were not mature, with follow-up ongoing. Additional studies are evaluating ixazomib maintenance in combination with existing agents. For example, a phase 2 study has demonstrated the feasibility and activity of long-term ixazomib-lenalidomide therapy as post-ASCT maintenance^59^. The doublet improved responses in 45% of patients, and median PFS has not been reached after a median follow-up of >3 years^59^. Only 6% of patients discontinued ixazomib due to toxicity^59^, providing further evidence for its feasibility as a component of long-term treatment approaches.

### Optimal duration of post-ASCT maintenance

An outstanding question in post-ASCT maintenance is regarding optimal duration of treatment. The studies included in a meta-analysis of lenalidomide maintenance in key phase 3 trials (in which the mean treatment duration was 28 months) were all of the treat-to-progression approach^32,34,36,37^. However, there are other trials that use a fixed-duration approach for 1−2 years^42,60^. Comparative studies of these approaches, and of fixed-duration maintenance vs. placebo, are not available. However, it is important to balance potential benefits and risks. Some patients may derive an optimal benefit/risk balance from shorter-term/fixed-duration lenalidomide maintenance (similar to findings with thalidomide maintenance of relatively limited duration^26,28,30^), whereas in other settings a longer treatment duration may be warranted. For example, in the phase 3 GMMG-MM5 trial (Table 3), patients received lenalidomide maintenance post ASCT for either 2 years or until they achieved CR^60^. No significant difference in PFS was seen between groups but 3-year OS rates were significantly higher in the 2-year treatment group. However, this was accompanied by a significant increase in toxicity. Nevertheless, results suggest that lenalidomide maintenance beyond CR achievement offers improved outcomes^60^. Similar conclusions have been reported from a pooled analysis in the post-ASCT and nontransplant settings, which demonstrated prolonged survival with maintenance vs. no maintenance in patients achieving a CR post-induction/consolidation, thereby indicating the importance of continuing treatment in these patients^61^. To date, fixed-duration approaches have been used in studies of proteasome inhibitor-based maintenance^53,54,58^, leaving the question of whether longer treatment might have further improved outcomes.

In this context, a follow-up question might be: at what depth of response might maintenance be stopped without affecting outcomes? The potential utility of MRD status for determining use and/or duration of maintenance therapy has been reviewed previously and potential study designs have been suggested to evaluate whether MRD-negative patients require ongoing therapy^13^. Data from Myeloma XI showed a PFS advantage with lenalidomide maintenance regardless of MRD status and demonstrated an increased rate of conversion from MRD-positive to MRD-negative status with lenalidomide (32%) vs. observation (4%)^44^. Preliminary data from another study suggest that MRD-negative status conferred high PFS values regardless of lenalidomide maintenance use (2-year PFS 88% vs. 74%), whereas in MRD-positive patients lenalidomide vs. no maintenance resulted in a significantly higher 2-year PFS rate (94% vs. 45%)^62^. In TOURMALINE-MM3, median PFS with ixazomib vs. placebo maintenance was 38.6 vs. 32.5 months (HR 0.61) in patients who were MRD-negative at study entry and 23.1 vs. 18.5 months (HR 0.70) in MRD-positive patients^58^. Further investigation is warranted, utilizing increasingly sensitive MRD assessment techniques, to determine whether MRD status can guide duration of post-ASCT maintenance—and of continuous therapy more broadly^13^—with an increasing number of trials demonstrating high rates of MRD-negativity (e.g. with lenalidomide maintenance^24,44,46^) and incorporating MRD status as a clinical and regulatory endpoint (Table 4).

**Table 4:** Ongoing phase 3 and randomized phase 2 comparative studies of continuous therapy and maintenance treatment approaches that have not yet reported data at the time of publication (ClinicalTrials.gov, April 26, 2019).

Study	NCT number	Phase	Maintenance/continuous treatment regimens	*N*	Primary endpoint	Estimated 1° completion date
Post-ASCT maintenance therapy						
GEM2014MAIN	NCT02406144	3	Ixazomib-Rd vs. Rd	316	PFS	Not known
MMRC	NCT02253316	2	Ixazomib vs. R	240	MRD	November 2019
NCI-2015-00138	NCT02389517	2	Ixazomib-Rd vs. R	86	MRD	March 2020
ATLAS	NCT02659293	3	Carfilzomib-Rd vs. R	180	PFS	March 2019
FORTE	NCT02203643	2	Carfilzomib-R vs. R	477	≥VGPR rate post-induction	October 2016^a^
Cassiopeia	NCT02541383	3	Daratumumab vs. observation	1085	PFS	August 2022
EMN18^b^	NCT03896737	2	Daratumumab-ixazomib vs. ixazomib	400	MRD-neg rate; 2-year PFS	February 2022
AURIGA/MMY3021	NCT03901963	3	Daratumumab-R vs. R	214	MRD-neg rate at 12 months	May 2021
GRIFFIN/MMY2004	NCT02874742	2	Daratumumab-R vs. R	222	sCR rate post-consolidation	January 2019
DraMMatic^c^	SWOG1803/BMT CTN 1706	3	Daratumumab-R vs. R	Not known	Not known	Not known
GMMG-HD6	NCT02495922	3	Elotuzumab-R vs. R	564	PFS	June 2020
GMMG-HD7	NCT03617731	3	Isatuximab-R vs. R	662	PFS	May 2025
Continuous frontline therapy, non-ASCT setting						
TOURMALINE-MM2	NCT01850524	3	Ixazomib-Rd vs. placebo-Rd	701	PFS	February 2018
COBRA	NCT03729804	3	Carfilzomib-Rd vs. VRd	250	PFS	December 2021
GEM2017FIT	NCT03742297	3	Daratumumab + carfilzomib-Rd vs. carfilzomib-Rd vs. VMP-Rd	300	CR rate	October 2020
Perseus	NCT03710603	3	Daratumumab-VRd–daratumumab-R vs. VRd–R	690	PFS	May 2029
MMY3019	NCT03652064	3	Daratumumab-VRd–daratumumab-Rd vs. VRd–Rd	360	MRD-neg rate	March 2024
ELOQUENT-1	NCT01335399	3	Elotuzumab-Rd vs. Rd	750	PFS	May 2019
SWOG S1211	NCT01668719	2	Elotuzumab-VRd vs. VRd	122	PFS	May 2019
IMROZ	NCT03319667	3	Isatuximab-VRd–isatuximab-Rd vs. VRd–Rd	440	PFS	December 2022
Post-induction maintenance therapy, non-ASCT setting						
TOURMALINE-MM4 + China continuation	NCT02312258 NCT03748953	3	Ixazomib vs. placebo	706 105	PFS	August 2019 September 2024
Myeloma XIV (FiTNEss)	NCT03720041	3	Ixazomib-R vs. placebo-R (post-ixazomib-Rd)	740	PFS	December 2024
X16108	NCT03733691	2	Ixazomib-R vs. ixazomib	52	PFS, AEs	December 2023
AGMT_MM-2	NCT02891811	2	Carfilzomib vs. observation	146	Post-induction ORR	September 2023

*AEs* adverse events, *ASCT* autologous stem cell transplant, *CR* complete response, *MRD-neg* negative for minimal residual disease, *ORR* overall response rate, *PFS* progression-free survival, *R* lenalidomide, *Rd* lenalidomide-dexamethasone, *VMP* bortezomib-melphalan-prednisone, *VRd* bortezomib-lenalidomide-dexamethasone.

^a^Data reported from induction/consolidation phase^63^; data not yet reported from the randomized maintenance phase of the study.

^b^Includes information from https://www.myeloma-europe.org/trials/emn-18/.

^c^Information from https://www.swog.org/clinical-trials/s1803.

### Ongoing randomized comparative studies

There are several ongoing randomized comparative studies yet to report data that are addressing the specific impact of newer agents within the post-ASCT maintenance setting (Table 4). The GEM2014MAIN study is evaluating addition of ixazomib to lenalidomide-dexamethasone (Rd) as maintenance, while the phase 3 ATLAS and FORTE studies are assessing carfilzomib-R(d) vs. lenalidomide in this setting, with data from the induction/consolidation phase of FORTE having already been reported^63^. The Cassiopeia study includes post-ASCT randomization to daratumumab maintenance vs. observation, the EMN18 study is evaluating addition of daratumumab to ixazomib maintenance, while daratumumab, elotuzumab, and isatuximab are being studied in combination with lenalidomide as post-ASCT maintenance in studies by the SouthWest Oncology Group (SWOG) and the German-speaking Multicenter Myeloma Group.

## Continuous frontline therapy in the nontransplant setting

### Current treatment approaches

Since initial publication of the phase 3 FIRST trial^64^, continuous Rd has emerged as a standard-of-care frontline therapy, with other continuous treatment regimens building upon this doublet. FIRST evaluated the outcome benefits of continuous Rd vs. fixed-duration Rd for 18 cycles (Rd18) vs. fixed-duration melphalan-prednisone-thalidomide (MPT)^64^. At the initial analysis, PFS was improved with continuous Rd vs. Rd18 and vs. MPT, response rates were higher with continuous Rd and Rd18 vs. MPT, and OS rates were higher with continuous Rd vs. MPT (Table 5)^64^. The subsequent final analysis confirmed these findings—the 4-year PFS rate with continuous Rd was more than double those with Rd18 and MPT; furthermore, there was a significant OS benefit with continuous Rd vs. MPT, although OS was similar with continuous Rd and Rd18^4^.

**Table 5:** Summary of data from key phase 3 studies of continuous therapy in the nontransplant setting.

Study	Treatment	*N*	Follow-up	DoT	Key efficacy outcomes	Key safety and tolerability data
FIRST^4,64^	Continuous Rd vs. Rd (18 cycles) vs. MPT (72 weeks)	535 vs. 541 vs. 547	Initial analysis: 37.0 mos	Median: 18.4 vs. 16.6 vs. 15.4 mos	ORR: 75% vs. 73% vs. 62% ≥ VGPR: 43% vs. 42% vs. 28% Median PFS: 25.5 vs. 20.7 vs. 21.2 mos (HR 0.70 vs. Rd18/0.72 vs. MPT) 4-yr OS: 59% vs. 56% vs. 51% (HR 0.90 vs. Rd18/0.78 vs. MPT)	Grade 3/4 AEs: 85% vs. 80% vs. 89% Grade 3/4 infection: 29% vs. 22% vs. 17% SPMs: 3% vs. 6% vs. 5%
			Updated analysis: 67 mos	Mean: 25.5 vs. 12.6 vs. 11.9 mos	ORR: 81% vs. 79% vs. 67% ≥ VGPR: 48% vs. 47% vs. 30% 4-yr PFS: 32.6% vs. 14.3% vs. 13.6% (HR 0.70/0.69) Median OS: 59.1 vs. 62.3 vs. 49.1 mos (HR 1.02/0.78)	Grade 3/4 infection: 32% vs. 22% vs. 17% SPMs: 7% vs. 7% vs. 9%
SWOG S0777^69,70^	VRd-Rd vs. Rd (Rd to PD)	264 vs. 261	Initial analysis: 54 vs. 56 mos	NR	ORR: 82% vs. 72% ≥ VGPR: 43.5% vs. 31.8% Median PFS: 43 vs. 30 mos (HR 0.712) Median OS: 75 vs. 64 mos (HR 0.709)	Grade 3/4 AEs: 82% vs. 75% Grade ≥ 3 neurotoxicity: 33% vs. 11% Discontinuation due to AEs: 23% vs. 10% SPMs: 4% vs. 4%
			Updated analysis: 84 mos	17.4 mos (Rd post-induction)	≥ VGPR: 74.9% vs. 53.7% Median PFS: 41 vs. 29 mos (HR 0.742) Median OS: not reached vs. 69 mos (HR 0.709)	SPMs: 8% vs. 7%
RV-MM-PI-0752^67^	Rd-R vs. continuous Rd	98 vs. 101	25 mos	NR	ORR: 73% vs. 63% ≥ VGPR: 43% vs. 35% Median EFS: 9.3 vs. 6.6 mos (HR 0.72) Median PFS: 18.3 vs. 15.5 mos (HR 0.93) 18-mo OS: 85% vs. 81% (HR 0.73)	Dose reductions (9 cycles): R: 1% vs. 21% Dex: 17% vs. 29%
MAIA^68^	Dara-Rd vs. Rd	368 vs. 369	28 mos	25.3 vs. 21.3 mos	≥ VGPR: 79.3% vs. 53.1% ≥ CR: 47.6% vs. 24.9% Median PFS: not reached vs. 31.9 mos (HR 0.56) OS: 16.8% vs. 20.6% of patients had died; median not reached on either arm	Common grade 3/4 AEs: neutropenia (50.0% vs. 35.3%), anemia (11.8% vs. 19.7%), lymphopenia (15.1% vs. 10.7%), pneumonia (13.7% vs. 7.9%) Infections: any-grade, 86.3% vs. 73.4%, grade 3/4 32.1% vs. 23.3% Infusion-related reactions (Dara-Rd): 40.9% (2.7% grade 3/4) Discontinuation due to AEs: 7.1% vs. 15.9%

*AE* adverse event, *CR* complete response, *dara* daratumumab, *dex* dexamethasone, *DoT* duration of treatment, *EFS* event-free survival, *HR* hazard ratio, *mos* months, *MPT* melphalan-prednisone-thalidomide, *NR* not reported, *ORR* overall response rate, *OS* overall survival, *PD* progressive disease, *PFS* progression-free survival, *R* lenalidomide, *Rd* lenalidomide-dexamethasone, *SPM* second primary malignancy, *SWOG* Southwest Oncology Group, *VGPR* very good partial response, *VRd* bortezomib-lenalidomide-dexamethasone.

The benefit of continuous Rd vs. MPT has been demonstrated in multiple patient subgroups^4^, including those achieving CR, ≥VGPR, and ≥PR^65^, those with no, mild, or moderate renal impairment^66^, and those aged ≤75 or >75 years^4^. However, recently reported data from the RV-MM-PI-0752 study comparing continuous Rd with Rd followed by lenalidomide maintenance (Rd-R) in elderly and intermediate-fit NDMM patients showed no significant differences in efficacy between regimens but lower rates of adverse events (AEs) and dose reductions in the Rd-R arm (Table 5)^67^. These findings suggest that continuous Rd may not represent an optimal approach for these patients due to tolerability issues associated with long-term use of both agents, and a frailty-adjusted approach to long-term treatment of NDMM is needed.

In FIRST, continuous Rd did not appear to offer consistent benefit vs. MPT according to cytogenetic risk status^4^. Among patients with standard-risk cytogenetic abnormalities, there was a significant PFS (HR 0.66) and OS (HR 0.69) benefit with continuous Rd, but patients with high-risk cytogenetics had similar outcomes with each therapy (PFS HR 1.27, OS HR 0.92)^4^. The authors suggested that triplet regimens built upon the continuous Rd backbone may be required in high-risk patients.

The phase 3 MAIA study of daratumumab-Rd vs. Rd to progression recently demonstrated the feasibility and activity of such a continuous triplet therapy (Table 5)^68^. In the initial analysis, daratumumab-Rd resulted in a significant 44% reduction in risk of progression or death, and response rates were significantly higher. Median OS was not reached in either arm; additional follow-up is required to evaluate long-term tolerability and efficacy.

The SWOG S0777 study also demonstrated the benefit of a triplet regimen (VRd) vs. Rd in the NDMM setting^69,70^; however, unlike in MAIA, VRd was administered for only eight cycles before patients discontinued bortezomib and continued Rd until progression. Nevertheless, the approach resulted in significant improvements in PFS and OS at the initial analysis that were maintained at an updated analysis after a median follow-up of 7 years (Table 5). Of note, median PFS was 38 vs. 16 months in the subgroup of 44 patients with high-risk disease by FISH, although this difference was not significant. The triplet appeared less tolerable than Rd, with substantially higher rates of grade ≥3 neurotoxicity and discontinuations due to AEs associated with the eight cycles of bortezomib therapy^69^; this could have been due to the use of intravenous instead of subcutaneous bortezomib, which has since become standard. Further studies are necessary to determine whether prolonged proteasome inhibitor therapy in addition to Rd could further improve outcomes.

A network meta-analysis in the setting of nontransplant NDMM has reinforced the findings from individual studies described above, albeit recent findings from MAIA were not included^71^. The analysis included studies of continuous Rd and VRd, and approaches utilizing a finite treatment duration or a post-induction maintenance approach (see next section). It found that, among approved treatment options, continuous Rd offered superior PFS and OS, and that among emerging treatment options only VRd resulted in significant improvements vs. continuous Rd^71^.

### Ongoing randomized comparative studies

Several randomized comparative studies of continuous triplet and quadruplet therapies are ongoing (Table 4). The benefit of adding ixazomib or elotuzumab to Rd is being investigated in the TOURMALINE-MM2 and ELOQUENT-1 studies, respectively; carfilzomib-Rd is being compared with VRd in the head-to-head COBRA study, and daratumumab and isatuximab are being investigated in combination with a VRd/Rd or carfilzomib-Rd backbone. These quadruplet regimens are being investigated primarily in younger and/or fitter patients, and, if tolerable, may offer substantial rates of sustained MRD-negativity and prolonged outcomes.

## Maintenance therapy post-induction in the nontransplant setting

In addition to continuous Rd being used alone and as a backbone for other long-term treatment, lenalidomide has been investigated as post-induction maintenance therapy, most commonly in “continuous lenalidomide” schemas involving lenalidomide-based induction followed by single-agent lenalidomide maintenance. Other agents, including thalidomide, bortezomib, ixazomib, and daratumumab, have been similarly studied in this setting (Table 6).

**Table 6:** Summary of data from key phase 3/randomized phase 2 studies of maintenance treatment post-induction in the nontransplant setting (data shown for overall treatment, including maintenance, and/or solely for maintenance phase where available).

Study	Treatment (maintenance dose/duration)	*N*	Follow-up	DoT	Key efficacy outcomes	Key safety and tolerability data
Myeloma XI^25,40^	R (10/25 mg, d 1–21, 28-d cycles, to PD) vs. observation post-CTD/CRD	407 vs. 316	31 months^a^	18 cycles (4-week cycles)^a^	Cumulative rate of response improvement at 60 months: 17.5% vs. 3.2% Median PFS: 26 vs. 11 months (HR 0.44) Median PFS2: 43 vs. 35 months (HR 0.72) 3-year OS: 66.8% vs. 69.8% (HR 1.02)	Grade 3/4 neutropenia: 28%/5%^a^ Discontinuations due to AEs: 28%^a^ SPMs: 5.3% vs. 3.1%^a^
MM-015^72^	MPR-R (10 mg, d 1–21, 28-d cycles, to PD) vs. MPR-placebo (d 1–21, 28-d cycles, to PD) vs. MP-placebo (d 1–21, 28-d cycles, to PD)	152 vs. 153 vs. 154	30 months	NR	Data from start of treatment: ORR: 77% vs. 68% vs. 50% ≥ VGPR: 32.9% vs. 32.7% vs. 12.3% Median PFS: 31 vs. 14 (HR 0.49) vs. 13 (HR 0.40) months 3-year OS: 70% vs. 62% vs. 66% Post-induction: Median PFS, MPR-R vs. MPR-placebo: 26 vs. 7 months (HR 0.34)	Data from start of treatment: Grade 4 neutropenia: 35% vs. 32% vs. 8% Grade 4 thrombocytopenia: 11% vs. 12% vs. 4% Discontinuations due to AEs: 16% vs. 14% vs. 5% SPMs: 7% vs. 7% vs. 3% Post-induction, MPR-R arm: Grade 4 neutropenia: 2% Grade 4 thrombocytopenia: 6% Grade 3/4 infection: 3%/2% Discontinuations due to AEs: 8%
GIMEMA RV-MM-PI-209^37^	R (10 mg, d 1–21, 28-d cycles, to PD) vs. no maintenance post-MPR (*n* = 132) or ASCT (*n* = 141) consolidation	126 vs. 125 (59 vs. 57 post-MPR)	51.2 months from enrollment	NR	MPR-R vs. MPR: Median PFS: 34.2 vs. 21.8 months 5-year OS: 70.2% vs. 58.7% R maintenance CR rate improvement: 20.0% to 33.8% R vs. no maintenance, post-MPR/ASCT Median PFS: 41.9 vs. 21.6 months (HR 0.47) OS: HR 0.64	R vs. no maintenance, post-MPR/ASCT Grade 3/4 AEs: Neutropenia 23.3% vs. 0% Infections 6.0% vs. 1.7% Dermatologic events 4.3% vs. 0% Discontinuation due to AEs: 5.2% vs. 0%
HOVON87/NMSG18^73^	MPT-T (100 mg/d to PD) vs. MPR-R (10 mg, d 1–21, 28-d cycles, to PD)	318 vs. 319	36 months	T vs. R maintenance: median 5 vs. 17 months	ORR: 81% vs. 84% ≥ VGPR: 47% vs. 45% Response improvement during maintenance: 23% vs. 18% Median PFS: 20 vs. 23 months (HR 0.87) 4-year OS: 52% vs. 56% (HR 0.82)	Grade 3/4 neutropenia: 27% vs. 64% Grade 3/4 thrombocytopenia: 8% vs. 30% Grade 3/4 neuropathy: 16% vs. 2% Discontinuation of maintenance due to AEs: 60% vs. 17% SPMs: 7% vs. 6%
ECOG E1A06^74^	MPT-T (100 mg/d to PD) vs. mPR-R (10 mg, d 1–21, 28-d cycles, to PD)	154 vs. 152	40.7 months	15.6 vs. 14.9 months 13.5 vs. 13.3 months of maintenance	ORR: 63.6% vs. 59.9% Median PFS: 21.0 vs. 18.7 months (HR 0.84) Median OS: 52.6 vs. 47.7 months (HR 0.88)	Grade ≥ 3 nonhematologic: Overall: 88% vs. 60% Maintenance: 19% vs. 11% Grade ≥ 4 hematologic: Overall: 61% vs. 49% Maintenance: 6% vs. 6% Overall discontinuation of maintenance due to AEs: 41.8% SPMs: 12.2% vs. 9.3%
NCT01091831^41^	RP (R 10 mg, d 1–21, 28-d cycles; P 50 mg, Q2d; to PD) vs. R alone post-CRD	57 vs. 49	41.0 vs. 42.3 months^b^	Median 28.9 vs. 25.3 months^b^	Data from enrollment (including CRD induction): Median PFS: 24.2 vs. 27.6 months 4-year OS: 68% vs. 76%	Grade 3/4 AEs:^b^ Neutropenia: 8% vs. 13% Infections: 8% vs. 5% Discontinuations due to AEs: 5% vs. 8%
GEM05MAS65^23,76^	VMP vs. VTP induction VT vs. VP maintenance (V 1.3 mg/m^2^ IV, d 1, 4, 8, 11, Q3M; T 50 mg/d; P 50 mg Q2d; up to 3 yrs)	130 vs. 130 91 vs. 87	72 months (maintenance 38 months)	NR	VMP vs. VTP plus maintenance: Median PFS: 32 vs. 23 months Median OS: 63 vs. 43 months (HR 0.67); 77 vs. 54 months in patients receiving maintenance Maintenance (VT vs. VP): CR rate increased from 24% to: 46% vs. 39% Depth of response improved in 19% Median PFS: 39 vs. 32 months 5-year OS: 69% vs. 50%	Maintenance (VT vs. VP): Grade 3/4 AEs: 17% vs. 5% Grade 3/4 PN: 9% vs. 3% Discontinuation due to AEs: 13% vs. 9%
GIMEMA-MM-03-05^77^	VMPT-VT (V 1.3 mg/m^2^ IV, Q2w; T 50 mg/d; up to 2 yrs) vs. VMP	254 vs. 257	23.2 months	82 patients received 6 months of VT	ORR: 89% vs. 81% ≥ VGPR: 59% vs. 50% CR: 38% vs. 24% 3-year PFS: 56% vs. 41% (HR 0.67) 3-year TTNT: 72% vs. 60% (HR 0.58) 3-year OS: 89% vs. 87% (HR 0.92) Response improvement during VT: ≥VGPR: 76% to 77% CR: 58% to 62%	Grade 3/4 neutropenia: 38% vs. 28% Grade 3/4 cardiologic AEs: 10% vs. 5% Grade 3/4 sensory PN: 8% vs. 5% Discontinuation due to AEs: 23% vs. 17% VT maintenance: Grade 3/4 AEs: 8% Grade 3 PN: 4%
UPFRONT^78^	V (1.6 mg/m^2^ IV, d 1, 8, 15, 22, 35-d cycles; up to five cycles) post-Vd vs VTD vs. VMP	168 vs. 167 vs 167 (maintenance: 82 vs. 60 vs. 69)	42.7 months	Median 8 vs. 6 vs. 7 cycles All five cycles of V maintenance: *n* = 53 vs. 43 vs. 53	ORR: 73% vs. 80% vs. 70% ≥ VGPR: 37% vs. 51% vs. 41% Median PFS: 14.7 vs. 15.4 vs. 17.3 months Median OS: 49.8 vs. 51.5 vs. 53.1 months Maintenance: Response improvement in 28 of 148 responding patients overall	Grade ≥ 3 AEs: 78% vs. 87% vs. 83% Grade ≥ 3 PN: 22% vs. 27% vs. 20% Maintenance: New-onset grade ≥ 3 PN: 6% vs. 7% vs. 3%
HOVON-126/NMSG21#13^86^	Ixazomib (4 mg, d 1, 8, 15, 28-d cycles, to PD) vs. placebo post-ITd	143; 39 vs. 39	26.4 months 18.6 months from rdz	NR	Post-induction: ORR: 81%; ≥ VGPR: 47%; CR: 9% Median PFS: 14.3 months 18-month OS: 85% From rdz: Response improvement: 10% vs. 13% Median PFS: 10.1 vs. 8.4 months 18-month OS: 100% vs. 92%	During induction: 8% PN Discontinuations due to toxicity: 17% During maintenance: No new-onset PN with ixazomib Discontinuations due to toxicity: 10% vs. 11%
ALCYONE^22,87^	Dara-VMP plus dara maintenance (16 mg/kg Q4w, with dex 20 mg, to PD) vs. VMP	350 vs. 356	Initial analysis: 16.5 months	14.7 vs. 12.0 months	ORR: 90.9% vs. 73.9% ≥ VGPR: 71.1% vs. 49.7% CR: 42.6% vs. 24.4% 18-month PFS: 71.6% vs. 50.2% (HR 0.50)	Grade 3/4 infections: 23.1% vs. 14.7% Dara infusion-related reactions: 27.7% SPMs: 2.3% vs. 2.5%
			Updated analysis: 27.8 months		≥ VGPR: 72.9% vs. 49.7% CR: 45.1% vs. 25.3% 2-year PFS: 63% vs. 36% (HR 0.43) 2-year PFS2: 84.1% vs. 78.5%	Grade 3/4 infections: 25.1% vs. 14.7% During dara maintenance: Grade 3/4 AEs: 23.7%

*AEs* adverse events, *ASCT* autologous stem cell transplant, *CR* complete response, *CRD* cyclophosphamide-lenalidomide-dexamethasone, *CTD* cyclophosphamide-thalidomide-dexamethasone, *d* day(s), *dara* daratumumab, *dex* dexamethasone, *DoT* duration of treatment, *HR* hazard ratio, *ITd* ixazomib-thalidomide-dexamethasone, *IV* intravenous, *maint* maintenance, *MP* melphalan-prednisone, *(m)MPR(-R)* (lower-dose) melphalan-prednisone-lenalidomide (plus lenalidomide maintenance), *MPT(-T)* melphalan-prednisone-thalidomide (plus thalidomide maintenance), *NR* not reported, *ORR* overall response rate, *OS* overall survival, *P* prednisone, *PD* progressive disease, *PFS* progression-free survival, *PFS2* progression-free survival from start of treatment to progression on next line of treatment, *PN* peripheral neuropathy, *Q2d* every other day, *Q2/4w* every 2/4 weeks, *Q3M* every 3 months, *R* lenalidomide, *rdz* randomization, *RP* lenalidomide-prednisone, *SPMs* second primary malignancies, *T* thalidomide, *TTNT* time to next therapy, *V* bortezomib, *Vd* bortezomib-dexamethasone, *VGPR* very good partial response, *VMP(T)* bortezomib-melphalan-prednisone-(thalidomide), *VP* bortezomib-prednisone, *VT* bortezomib-thalidomide, *VTD* bortezomib-thalidomide-dexamethasone, *VTP* bortezomib-thalidomide-prednisone, *yrs* years.

^a^Overall data in 1137 vs. 834 patients randomized to lenalidomide vs. observation across both the transplant-eligible and transplant-ineligible pathways.

^b^Overall data in 117 vs. 106 patients randomized to lenalidomide-prednisone vs. lenalidomide maintenance across the CRD and ASCT consolidation arms.

The efficacy of lenalidomide maintenance vs. observation post-lenalidomide/thalidomide-based induction in the nontransplant pathway of Myeloma XI has been reported recently^25,40^; results demonstrated a significant improvement in PFS and PFS2, but no OS benefit was seen (Table 6)^40^. Notably, the PFS benefit of lenalidomide maintenance was seen regardless of cytogenetic risk. Lenalidomide also improved depth of response in approximately one-fifth of patients^25^. Similarly, the MM-015^72^ and GIMEMA-RV-MM-PI-209^37^ studies have investigated the value of lenalidomide maintenance vs. placebo/observation following non-ASCT induction with melphalan-prednisone-lenalidomide (MPR) (Table 6). The “continuous lenalidomide” MPR-R schema resulted in significantly prolonged PFS compared to MPR induction alone in both studies; however, while a higher 5-year OS rate was seen with MPR-R in the GIMEMA RV-MM-PI-209 study, no significant OS benefit was reported for MPR-R vs. MPR-placebo or MP-placebo in MM-015. Of note, in MM-015 the PFS benefit with MPR-R was only seen in patients aged 65–75 years (median 31 vs. 15 months in MPR-placebo, vs. 12 months in the MP control arm) and not in patients aged >75 years, possibly associated with poorer tolerability of the triplet in this population; specifically, rates of grade 4 hematologic toxicities and discontinuations due to AEs were markedly higher with MPR vs. MP^72^. However, in a landmark analysis to isolate the activity of lenalidomide maintenance, there was a clear PFS benefit with continued lenalidomide therapy vs. placebo, both overall and regardless of age^72^. These findings further reinforce the importance of treatment tolerability with regards to overall feasibility of continuous therapy approaches, particularly for elderly and/or frail populations.

Additional phase 3 studies of continuous therapy approaches have compared MPR-R vs. MPT-T (Table 6)^73,74^. Due to their designs, these studies were not able to demonstrate the isolated benefit of post-induction maintenance with an immunomodulatory drug. Data from the HOVON87/NMSG18 and E1A06 trials showed no significant efficacy differences between the two regimens; however, there was greater toxicity in the thalidomide arms^73^. Other phase 3 studies, including EMN01^75^ and a European-Australian study^41^, have compared lenalidomide-prednisone to lenalidomide as maintenance following lenalidomide-based induction. Data specific to the impact of post-induction maintenance therapy have not been reported, but the European-Australian study showed similar PFS and OS from the start of induction with the two regimens, and an overall analysis of maintenance patients, including those receiving post-ASCT maintenance, showed similar rates of toxicity between lenalidomide-prednisone and lenalidomide^41^. The authors concluded that the advantage of adding steroids to immunomodulatory drugs during maintenance is unclear.

There have been no randomized phase 3 studies demonstrating the specific benefit of proteasome inhibitor-based or monoclonal antibody-based maintenance within this setting; however, proteasome inhibitor maintenance has been shown to be feasible and active following proteasome inhibitor-based induction. Bortezomib-based maintenance following bortezomib-based induction resulted in substantial increases in CR rate and contributed to lengthy outcomes in the GEM05MAS65 study^23,76^, and contributed to improved PFS vs. a no-maintenance approach in the GIMEMA-MM-03-05 study (Table 6)^77^. In the phase 3B community-based UPFRONT study, fixed-duration single-agent bortezomib maintenance following bortezomib-based induction improved response depth in approximately 16% of patients, with limited new-onset toxicity^78^. A phase 1/2 study has shown the feasibility and activity of carfilzomib maintenance following weekly carfilzomib-cyclophosphamide-dexamethasone induction, with response improvements again being reported^79^.

Similarly, single-agent ixazomib maintenance has been utilized following ixazomib-based induction in four phase 1/2 studies^45,80–83^; a pooled analysis of maintenance patients from these studies reported deepening responses in 23% of patients, as well as a median PFS of 21.4 months and a 3-year OS of 82% from the start of maintenance, with limited new-onset AEs^84^. Ixazomib-daratumumab-dexamethasone followed by ixazomib maintenance is being evaluated in unfit and frail patients in the phase 2 HOVON-143 study, which has reported promising safety and response data for the induction phase^85^. However, in the randomized phase 2 HOVON-126/NMSG21#13 trial, in which patients received ixazomib-thalidomide-dexamethasone induction and were then randomized to ixazomib or placebo maintenance^86^, preliminary data showed no response or PFS benefit with ixazomib maintenance to date, although ixazomib did not result in additional toxicity compared to placebo (Table 6). The ongoing phase 3 TOURMALINE-MM4 study will provide more comprehensive information on the use of single-agent ixazomib maintenance in the post-induction setting (Table 4).

The ALCYONE study has recently reported a substantial PFS benefit of continuous daratumumab treatment in the nontransplant setting, utilizing daratumumab-VMP plus daratumumab maintenance vs. VMP alone (Table 6)^22,87^. This long-term treatment approach has demonstrated PFS and PFS2 improvements vs. VMP, although OS data are not yet mature. Daratumumab maintenance was associated with a limited rate of grade 3/4 AEs^87^. Network meta-analyses in the non-ASCT setting^71,88^ and matched-pair patient analyses^89^ support the efficacy of daratumumab-VMP plus daratumumab maintenance vs. other treatment approaches. However, updated PFS curves suggest an increased rate of PFS events after 12 months in both arms^87^, following completion of the VMP component of therapy, suggesting the potential value of continuing proteasome inhibitor therapy with daratumumab maintenance.

## Safety and tolerability of long-term treatment approaches

Toxicity and treatment burden may limit treatment duration and drive patients’ preference for a treatment-free interval. Therefore, tolerability, limited treatment burden, absence of cumulative or chronic toxicity, and no adverse impact on QoL are important aspects for agents intended for continuous therapy or maintenance vs. fixed duration. The preceding sections have highlighted the substantial efficacy demonstrated by multiple agents in different treatment settings, but data from key studies (Tables 3, 5, 6) also show that regimens may be associated with safety and tolerability concerns that require consideration when selecting a long-term treatment approach. A recent retrospective study indicated no impact on PFS or OS of maintenance therapy with lenalidomide vs. bortezomib, the authors suggesting that side-effect profile and anticipated tolerability might be more valuable in guiding treatment choices^90^. However, patients were heterogeneously treated and for varying degrees of time beyond 2 years of maintenance; thus, findings of this retrospective analysis should be interpreted with caution.

The findings from phase 3 studies reviewed herein have highlighted the differential feasibility of some agents as long-term therapy, due to tolerability limiting treatment durations and increasing rates of discontinuation due to AEs (Table 3); indeed, for some agents/regimens, based on the benefit/risk balance, a fixed-duration approach may be more appropriate for some patients. Long-term therapy with both thalidomide^27,29,52,53,73^ and bortezomib^52,53,69,70^ has been associated with a substantially increased risk of peripheral neuropathy, which can be dose-limiting, while continuous and maintenance lenalidomide therapies have resulted in increased rates of hematologic toxicity, notably grade 3/4 neutropenia^25,40,73^, as well as chronic diarrhea^91^. Lenalidomide has also been associated with an increased risk of SPMs; in the meta-analysis of phase 3 studies of lenalidomide maintenance post ASCT, the cumulative rates of hematologic and solid SPMs prior to disease progression on lenalidomide maintenance were 5.3% and 5.8%, vs. 0.8% and 2.0% with placebo/observation^32^. However, as the authors highlight, this risk is outweighed by the significantly reduced risk of disease progression with lenalidomide maintenance.

Limited QoL data have been reported from studies of long-term treatment approaches; however, the available findings appear promising, suggesting that such therapies do not typically have an adverse impact on QoL (Table 7)^28,49,58,64,92–95^. However, commonly used QoL instruments may not capture all aspects of importance to patients receiving long-term therapy, e.g. sexual functioning. It is important to consider that some patients may not necessarily value a PFS benefit with long-term treatment approaches if not associated with better QoL or a treatment-free interval.

**Table 7:** QoL data reported from studies of long-term treatment approaches.

Study	Treatment	QoL instruments	Key QoL findings
Myeloma IX^92^	T (50–100 mg/day to PD) vs. no maintenance post ASCT	EORTC QLQ-C30/QLQ-MY24	•Minimal effects of thalidomide maintenance on various subscales •Small significant difference in favor of observation only for Global Health Status/QoL at 3 months (−3.39, *p* = 0.02) •No significant differences for Pain, Fatigue or Physical Functioning •Constipation worse with thalidomide maintenance vs. observation at 3 and 6 months
NCIC-CTG Myeloma 10^28^	TP (T 200 mg/d, P 50 mg Q2d; up to 4 years) vs. observation post ASCT	EORTC QLQ-C30 / trial-specific disease module	•QoL inferior with TP vs. observation for cognitive function domain and for symptoms of dyspnea, constipation, thirst, swelling in legs, numbness, dry mouth, and balance problems, reflecting the toxicity profile reported with this regimen •QoL scores improved with TP vs. observation for appetite and sleep
FIRST^64,93,94^	Continuous Rd vs. Rd (18 cycles) vs. MPT (72 weeks) QoL instruments were only administered at specific time-points up to and including cycle 18, plus at the end of the study. Thus, it was not feasible to compare QoL between the continuous Rd and Rd18 treatment arms, as treatment was essentially the same through the QoL data collection period	EORTC QLQ-C30/QLQ-MY20/EQ-5D	•Consistent positive impact on patients’ QoL with long-term Rd, with improvements from baseline through 18 months reported across subscales with continuous Rd/Rd18 •Significant improvements from baseline in all arms for Pain, Disease Symptoms, Global Health Status, Physical Functioning, EQ-5D Health Utility, and Fatigue •Rd showed clinically meaningful improvements in Pain domain, vs. none with MPT •Rd showed a significantly greater reduction in Disease Symptoms vs. MPT at month 3 •Treatment Side Effects domain worsened from baseline in all arms, but scores were significantly better with Rd vs. MPT •Predicted QoL scores beyond 18 months of Rd suggested that QoL improvements were maintained or improved
Phase 3 LenaMain study^95^	R 25 mg vs. R 5 mg (to PD) following 6 months of post-ASCT consolidation	EORTC QLQ-C30	•No overall adverse impact on QoL subscales with either maintenance dose •Mean change in Global Health Status/QoL of –4 vs. –8 after 2 years •Trend for better overall QoL in the higher-dose arm, including significantly better role functioning, but with a significantly greater increase from baseline in diarrhea symptom score •Further illustrating the importance of evaluating benefit/risk balance, the 25 mg dose was associated with significantly longer event-free survival but a 10% increase in grade 3/4 infections per year
Connect® MM registry^49^	R maintenance vs any maintenance vs. no maintenance	FACT-MM, EQ-5D, BPI	•No adverse impact on QoL of lenalidomide or any maintenance compared to no maintenance •FACT-MM, EQ-5D, and BPI scores improved post ASCT in all groups, with no significant differences in change from baseline
TOURMALINE-MM3^58^	Ixazomib (3–4 mg, d 1, 8, 15, 28-d cycles; up to 2 years) vs. placebo post ASCT	EORTC QLQ-C30/QLQ-MY20	•No detrimental impact on patients’ QoL with ixazomib compared to placebo •Similar mean scores maintained from study entry to end of treatment in both groups, including for functioning, symptoms, and side-effects scales, except Nausea or Vomiting and Diarrhea, which were negatively affected in the ixazomib arm

*ASCT* autologous stem cell transplant, *BPI* Brief Pain Inventory, *EORTC* European Organisation for Research and Treatment of Cancer, *EQ* EuroQoL, *FACT* Functional Assessment of Cancer Therapy, *MPT* melphalan-prednisone-thalidomide, *P* prednisone, *PD* progressive disease, *Q2d* every other day, *QLQ* quality of life questionnaire, *QoL* quality of life, *R* lenalidomide, *Rd(18)* lenalidomide-dexamethasone (for 18 cycles), *T* thalidomide, *TP* thalidomide-prednisone.

The impact on patients of the burden of prolonged treatment also requires consideration. This treatment burden may arise due to the need for repeated trips for hospital or physician appointments, or for repeated intravenous or subcutaneous drug administrations^96^. These inconveniences of receiving treatment may limit the feasibility of continuous or maintenance therapy with some agents in the real-world setting^17^. As reviewed herein, studies suggest that prolonged treatment is associated with improved PFS, and so novel approaches may be required to enable patients to continue therapy for as long as possible to achieve this PFS benefit; for example, switching from a parenterally administered to an oral therapy may make prolonged proteasome inhibitor therapy more feasible in the community setting, an approach currently being explored with ixazomib in the ongoing US MM-6 trial^97^.

## Pharmacoeconomics of long-term treatment approaches

While long-term treatment approaches are associated with improved outcomes, they may potentially be associated with a substantial economic impact due to patients receiving novel agents for a period of several years. Conversely, however, there may be long-term economic benefit as the efficacy of these approaches may delay the need for subsequent therapy and may reduce the healthcare burden due to disease-related side-effects. The economic impact/benefit may depend on the time period being evaluated and on whether the long-term treatment approach is associated only with a PFS benefit or also with an OS benefit. Thus, pharmacoeconomic evaluation is important to the emerging paradigm of continuous therapy. A number of analyses related to lenalidomide maintenance therapy have already been reported (Table 8)^48,98–100^.

**Table 8:** Pharmacoeconomic analyses related to the use of lenalidomide maintenance therapy.

Study	Analysis	Data source	Treatment	Findings
Jackson et al.^98^	European (EU5) cost impact analysis	Cost-pathway model based on Myeloma XI dosing, real-world clinical prescribing, and expert clinical opinion	Lenalidomide maintenance (10 mg, assumed 50% received daily, 50% received d 1−21 in 28-d cycles; duration assumed per CALGB 100104 (Table 1)^34^) vs. no maintenance	Lower direct medical costs per patient over a 5-year period post ASCT (€209,600 vs. €276,900), attributed to a reduced requirement for subsequent lines of treatment
Connect® MM^48^	Analysis of healthcare resource utilization	NDMM patients in Connect® MM who received induction and single ASCT	Lenalidomide-only maintenance (*n* = 180), any maintenance (*n* = 256), or no maintenance (*n* = 165), dosing not defined, for up to 2 years	No increased rates of healthcare resource utilization, including similar hospitalization rates, with lenalidomide compared with no maintenance.
Zhou et al.^100^	Cost-effectiveness analysis, US payer perspective	Partitioned survival model based on data from CALGB 100104, pooled analysis of lenalidomide maintenance, and published literature	Lenalidomide maintenance (duration estimated per phase 3 meta-analysis (Table 1)^32^) vs. no maintenance and bortezomib maintenance (duration estimated from published literature)	Life-years gained: 3.64 and 2.76 QALYs gained: 2.99 and 2.42 Incremental costs per life-year: $130,817 and $149,411 Incremental costs per QALY: $159,240 and $170,408 (WTP threshold: $200,000)
Uyl-de-Groot et al.^99^	Cost-effectiveness analysis, Netherlands perspective	Partitioned survival model based on data from pooled meta-analysis of CALGB 100104, GIMEMA RV-MM-PI-209, and IFM 2005-02 studies; utility data from Connect® MM	Lenalidomide (10 mg, d 1–21, 28-d cycles; efficacy and safety from phase 3 meta-analysis (Table 1)^32^) vs. no maintenance	Life-years gained: 2.79 QALYs gained: 2.26 Cost increase (first line): €147,707 Overall cost increase: €71,536 Deterministic ICER: €31,695 (WTP threshold: €50,000)

*ASCT* autologous stem cell transplantation, *ICER* incremental cost-effectiveness ratio (cost/QALY), *QALY* quality-adjusted life-year, *WTP* willingness-to-pay.

## Conclusions

The paradigm of long-term treatment is becoming increasingly well-established in different treatment settings in NDMM, with data reported on multiple agents and regimens in each setting clearly demonstrating the value of prolonged treatment duration on PFS and, in some cases (primarily lenalidomide as post-ASCT maintenance), OS. Ongoing studies are anticipated to provide further confirmation of this PFS and possibly OS benefit over the coming 5 years and may result in the addition of numerous novel therapy options to the long-term treatment armamentarium, providing physicians with a greater selection of therapeutic pathways for patients.

In order to optimize individual patient outcomes, it will be important to elucidate which continuous therapy and maintenance treatment approaches are most appropriate for which patient subgroups, taking into account not only clinical efficacy and safety but also tolerability, QoL, and patients’ perspectives regarding the feasibility, convenience, and burden of long-term treatment. For example, some patients may prefer to maximize QoL and enjoy a treatment-free interval rather than have a PFS benefit with no QoL improvement with long-term therapy. To further inform such treatment decisions, longer follow-up of ongoing studies will be important to determine whether OS benefits are seen with long-term treatment approaches other than lenalidomide as post-ASCT maintenance. Ongoing studies of continuous/maintenance therapy with monoclonal antibodies may hold particular promise in this regard. Additional randomized comparisons of different treatment durations and intensities, such as between more intense fixed-duration and continuous approaches, would also be of value for determining optimal treatment duration in different patients; such evidence-based information is not currently available.

Various other clinically important questions remain to be answered regarding continuous therapy and maintenance, such as optimal treatment duration, dosing schedule, the potential role of MRD evaluation in guiding decisions regarding continuation of treatment (and potentially as a regulatory endpoint), and how best to tailor treatment duration and intensity in the context of patient age and fitness, in order to provide optimal outcomes. The data in this review and the breadth of ongoing phase 3 studies offer encouragement that further improvements in patient survival will result from these long-term treatment approaches, potentially transforming MM into a chronic condition for many patients.
